# Supplementary material for: Design of a Robust Flow Cytometric Approach for Phenotypical and Functional Analysis of Human Monocyte Subsets in Health and Disease
Source: Biomolecules. 2024 Oct 3;14(10):1251. doi: 10.3390/biom14101251 (PMC11506830; doi:10.3390/biom14101251)
Supplement: Supplementary file 1 [file biomolecules-14-01251-s001.zip › biomolecules-3165028-supplementary.pdf]

Supplemental Material

Design of a robust flow cytometric approach for phenotypical and functional analysis of human monocyte subsets in health and disease

Talia Ahrazoglu [1,†], Jennifer Isabel Kluczny [1,†], Patricia Kleimann [2], Lisa-Marie Irschfeld [3], Fabian Theodor Nienhaus [4], Florian Bönner [4], Norbert Gerdes [4,5] and Sebastian Temme [1,5,*]

[1] Department of Anesthesiology, Faculty of Medicine, University Hospital, Heinrich-Heine-University, 40225 Düsseldorf, Germany; talia.ahrazoglu@hhu.de (T.A.); jennifer.kluczny@hhu.de (J.-I.K.)

[2] Institute of Molecular Cardiology, Faculty of Medicine, University Hospital, Heinrich-Heine-University, 40225 Düsseldorf, Germany; patricia.kleimann@uni-duesseldorf.de

[3] Department of Radiation Oncology, Faculty of Medicine, University Hospital, Heinrich-Heine-University, 40225 Düsseldorf, Germany; lisa-marie.irschfeld@med.uni-duesseldorf.de

[4] Department of Cardiology, Pulmonology and Vascular Medicine, Faculty of Medicine, University Hospital, Heinrich-Heine University, 40225 Düsseldorf, Germany; fabiantheodor.nienhaus@med.uni-duesseldorf.de (F.T.N.); florian.boenner@krankenhaus-dueren.de (F.B.); gerdes@hhu.de (N.G.)

[5] Cardiovascular Research Institute Düsseldorf (CARID), Medical Faculty, Heinrich-Heine University, 40225 Düsseldorf, Germany

* Correspondence: sebastian.temme@uni-duesseldorf.de; Tel.: +49-211-81-05100

† These authors contributed equally to this work

Supplemental figures

Figure S1: Characterization of Perfluorocarbon nanoemulsions (PFCs)

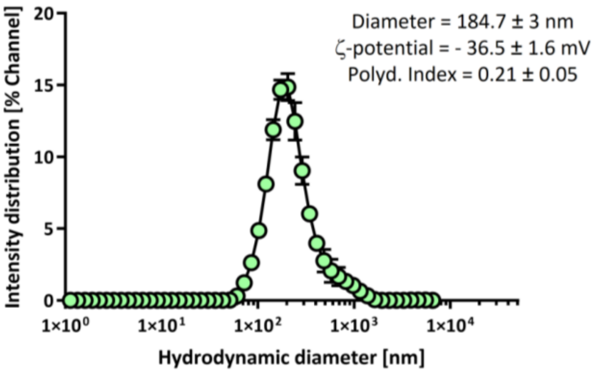

Figure S1: Dynamic light scattering (DLS) analysis of ^{A488}PFCEs: Fluorescently labelled (Atto488-DPPE, DPPE = 1,2-Dipalmitoyl-sn-glycero-3-phosphoethanolamine) PFCs were prepared by microfluidization with the lipid mixture E80S (see section 2.2 for details). Hydrodynamic diameter, polydispersity index, and ζ-potential were determined by DLS. PFCs were measured three times with ten cycles each. The graph shows the intensity weighted distribution of the DLS measurements. This revealed a mean value for the hydrodynamic diameter of 184.7 nm (± 3 nm). The polydispersity index as an indicator for size-distribution was 0.21 (± 0.05), while the ζ-potential was -36.5 mV (± 1.6 mV). Data are mean values ± SD of n = 3 sets of measurements (technical replicates).

Figure S2: Full gating of strategies A-C for identification of monocyte subsets

A Gating - Strategy A

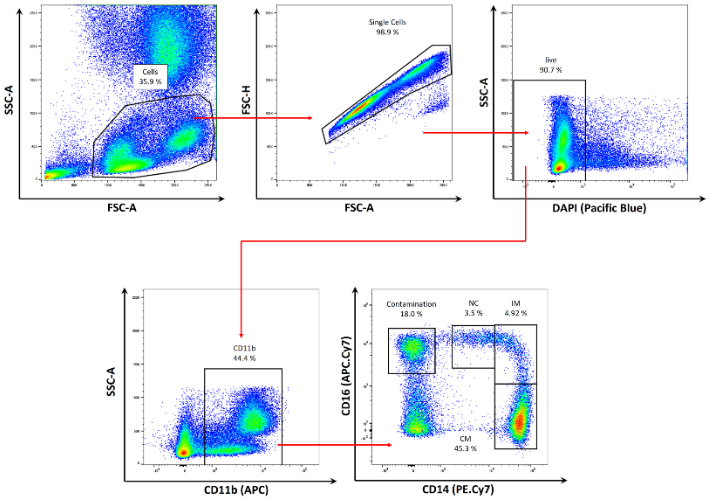

B Gating - Strategy B

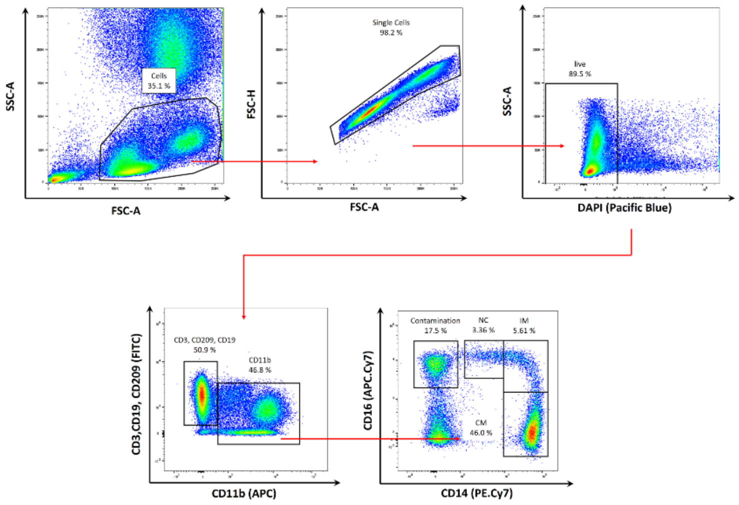

C Gating - Strategy C

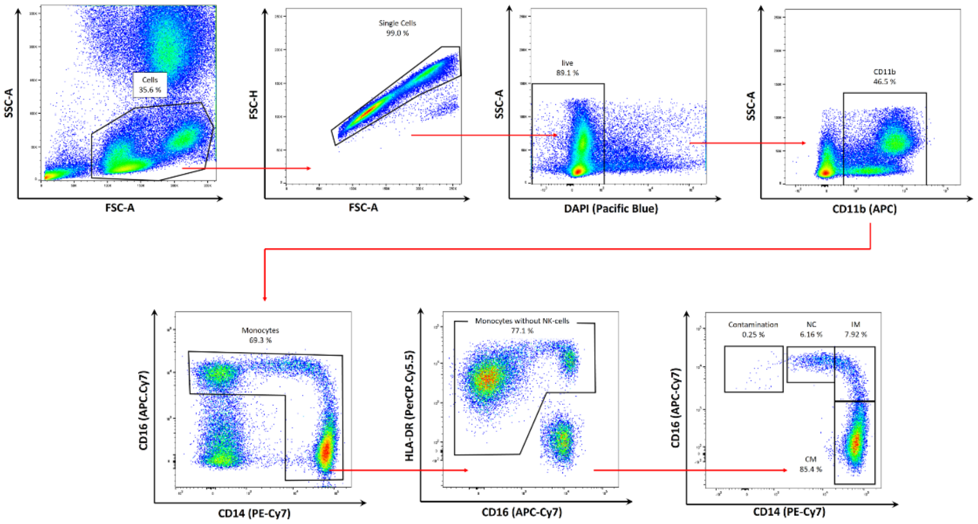

Figure S2: Full gating strategies for the identification of monocyte subsets by strategies A-C: Human whole blood was obtained from healthy volunteers, erythrocytes were lysed and cells were stained with: **A)** CD11b-APC, CD14-PE.Cy7, CD16-APC.Cy7 (Strategy A); **B)** CD11b-APC, CD14-PE.Cy7, CD16-APC.Cy7 and CD3-FITC, CD19-FITC, CD209-FITC (Strategy B); **C)** CD11b-APC, CD14-PE.Cy7, CD16-APC.Cy7, HLA-DR-PerCP.Cy5.5 (Strategy C). Blood cells were additionally stained with DAPI (4′,6-Diamidin-2-phenylindol) to exclude dead cells (DAPI^{+}) from the analysis. Cells were first gated based on their FSC/SSC properties, followed by exclusion of doublets and dead cells before they were selected based on the expression of cell surface molecules according to strategies A-C. CM = classical monocytes, IM = intermediate monocytes, NC = non-classical monocytes.

Figure S3: CD56 expression of monocyte subsets

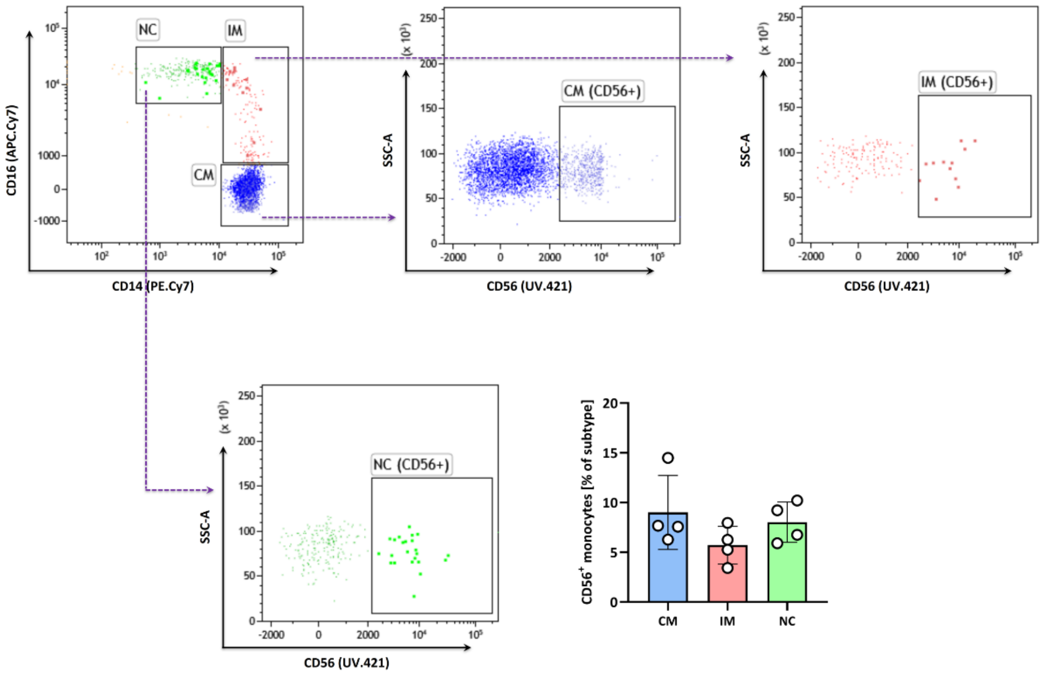

Figure S3: CD56 expression of monocyte subtypes: Blood immune cells from healthy volunteers were stained with antibodies against CD11b, HLA-DR, CD16, CD16 and CD56 and gated according to strategy C to identify monocyte subtypes (upper left). The CD56-expression of classical (CM, blue, upper middle), intermediate (IM, red, upper right) and non-classical (NC, green, lower left) monocytes is displayed. A quantification of the relative amount of $CD56^{+}$ cells revealed that between 5-10 % of all monocytes of young healthy volunteers express CD56. Data are mean values ± SD of n = 4 samples.

Figure S4: CD56 expression of CD16^{++}/CD14^{-} cells

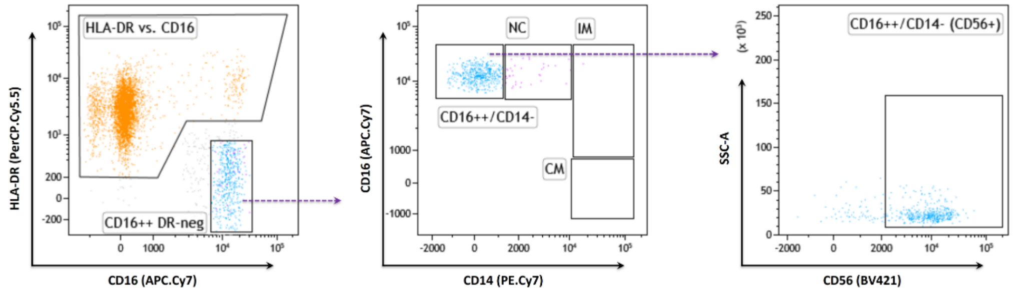

Figure S4: CD56 expression of the CD16^{++}/CD14^{-} population: Blood from healthy volunteers was subjected to erythrocyte lysis and the obtained cells were stained with antibodies against CD11b, HLA-DR, CD16, CD16 and CD56. Cells were first gated according to strategy C (not shown). Because NK cells do not express MHC-class II molecules, the CD16^{++}/HLA-DR negative population (left) was selected and plotted against CD14/CD16 (middle). This CD16^{++}/HLA-DR^{-}/CD14^{-} expresses high levels of CD56 (right), which is characteristic for NK cells.

Figure S5: **FSC/SSC properties of monocyte subpopulations**

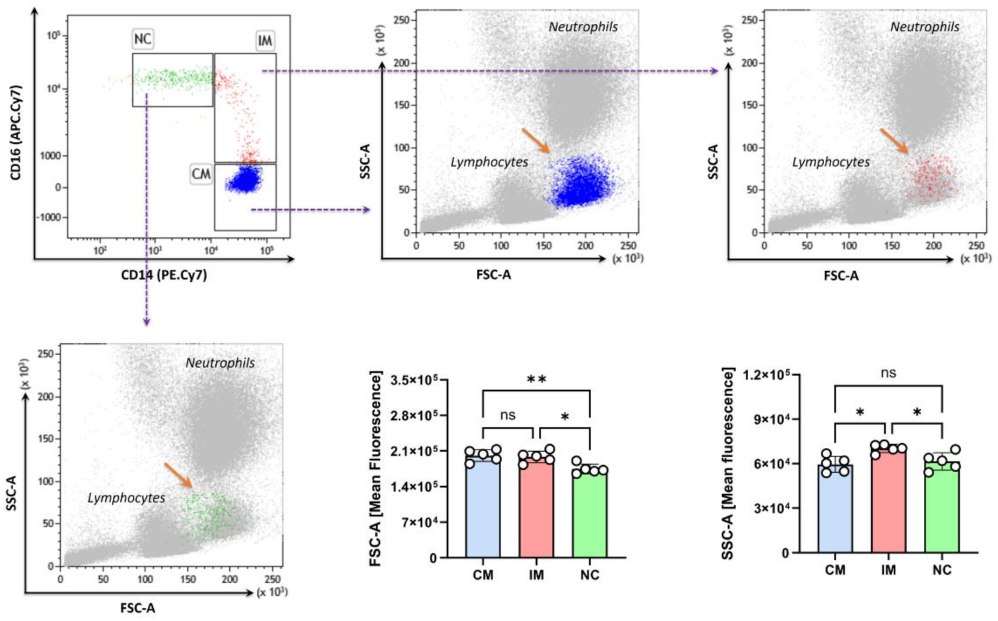

Figure S5: FSC/SSC properties of monocyte subpopulations: Blood was obtained from young healthy volunteers and after lysis of the erythrocytes, blood immune cells were stained and gated according to strategy C. To this end, cells were incubated with antibodies against CD11b, CD14, CD16, HLA-DR to identify monocyte subtypes and DAPI to exclude dead cells. The left upper plot shows the identification of monocytes subtypes based on the expression of CD14 and CD16. NC = nonclassical monocytes (green); IM = intermediate monocytes (red); CM = classical monocytes (blue). CM, IM and NC were subjected to back-gating to show their localization in the initial FSC/SSC plot. Note that the NC monocyte population shows a reduced FSC that partially overlaps with the lymphocyte population. Lower middle and right: Quantification of the FSC-A (middle) and SSC-A (right) values of n = 5 independent blood samples. Displayed are the mean values ± SD. Normality was tested using a Shapiro-Wilk test and variability with Levene´s test. Differences between the groups were analyzed by a one-way ANOVA followed by Turkey´s multiple comparisons test. Statistical significance: * = $p \leq 0.05$, ** = $p \leq 0.01$, ns = not significant.

Figure S6: Full gating strategy for healthy volunteers and CHD and STEMI patients

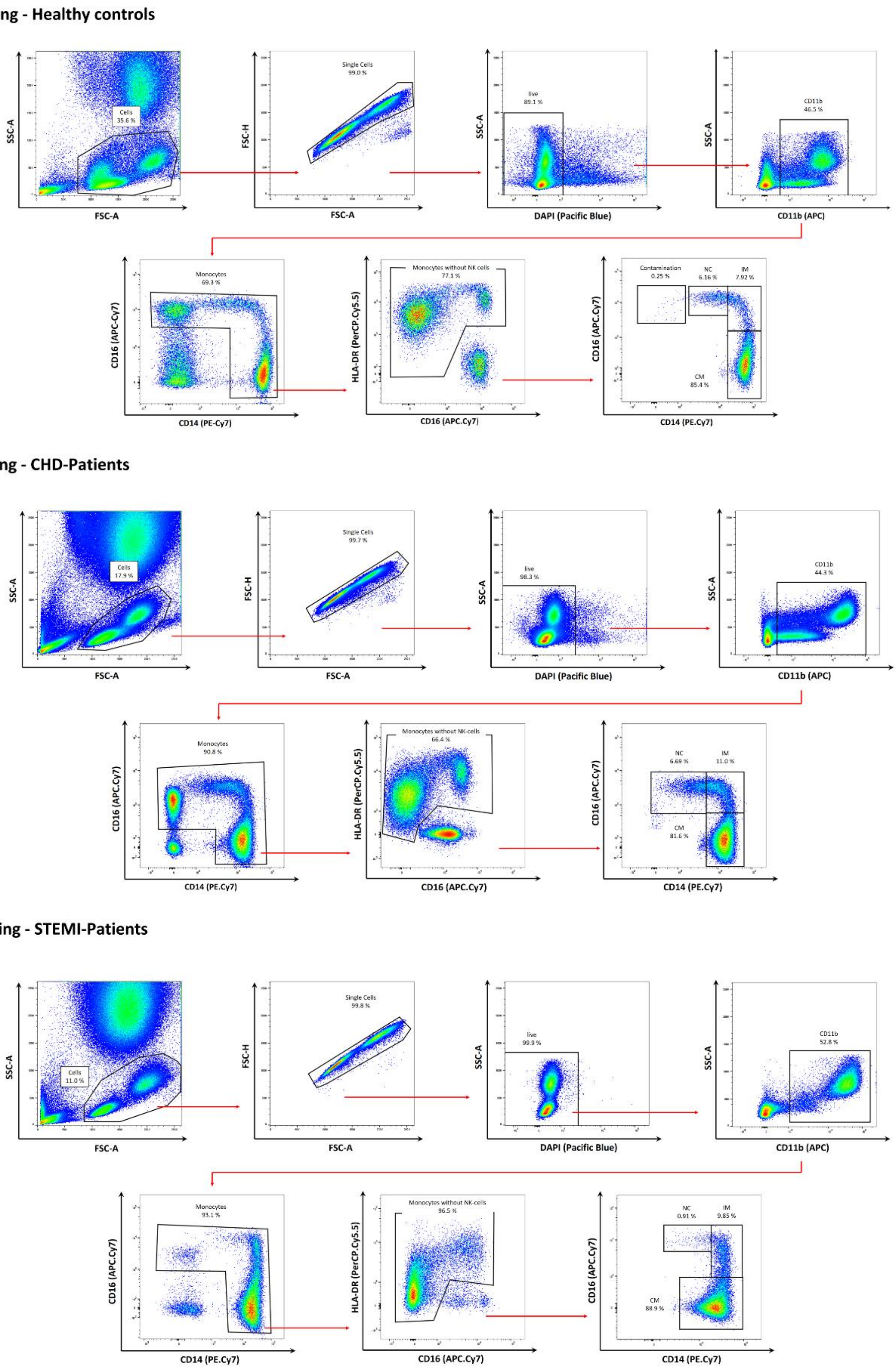

Figure S6: Full gating strategy for healthy volunteers and CHD and STEMI patients: Blood samples derived from healthy volunteers **(A)**, patients with stable coronary heart disease (CHD) **(B)**, and ST-elevation myocardial infarction (STEMI) **(C).** After erythrocyte lysis, cells were stained against CD11b,

CD14, CD16 and HLA-DR (= Strategy C) to identify monocyte subsets. Displayed are pseudocolor plots that show the whole gating strategy, including doublet exclusion (second plot of each row), alive-dead differentiation using DAPI (third plot of each row) and the identification of the monocyte subtypes based on the expression of CD11b, HLA-DR, CD14 and CD16.

Figure S7: Monocyte identification after improper storage

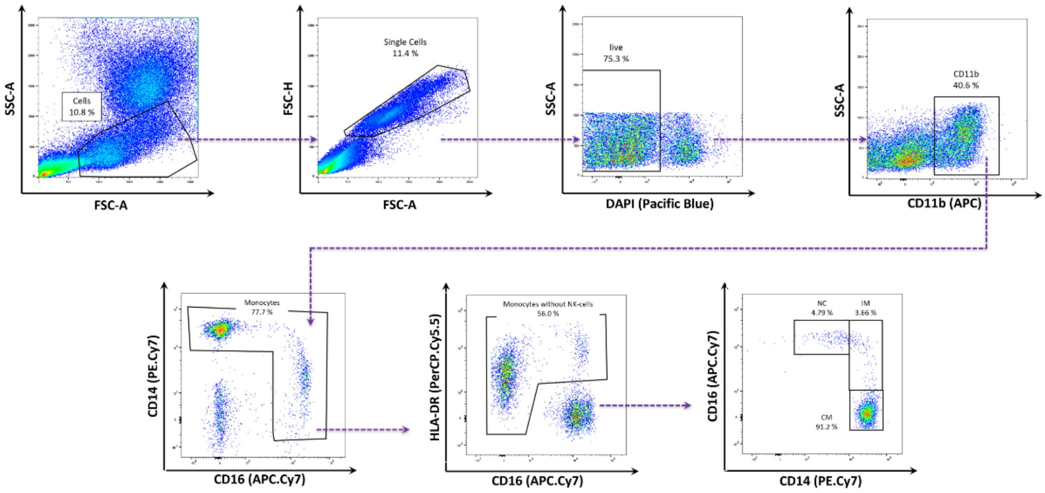

Figure S7: Flow cytometric analysis of monocyte subpopulations after improper storage: Monocytes from healthy volunteers were stored at room temperature for one hour and subsequently processed for flow cytometric analysis. Erythrocytes were lysed and the cells were incubated with antibodies that bind to CD11b, HLA-DR, CD14 and CD16 (Strategy C). DAPI staining was conducted to exclude dead cells ($DAPI^+$) from the analysis. Shown are typical pseudocolor plots that show the sequential gating strategy to identify monocyte subpopulations based on the CD14/CD16 expression pattern (lower, right). CM = classical monocytes, IM = intermediate monocytes, NC = non-classical monocytes.

Figure S8: Expression of CCR2 and CX3CR1 in fixed and permeabilized monocytes

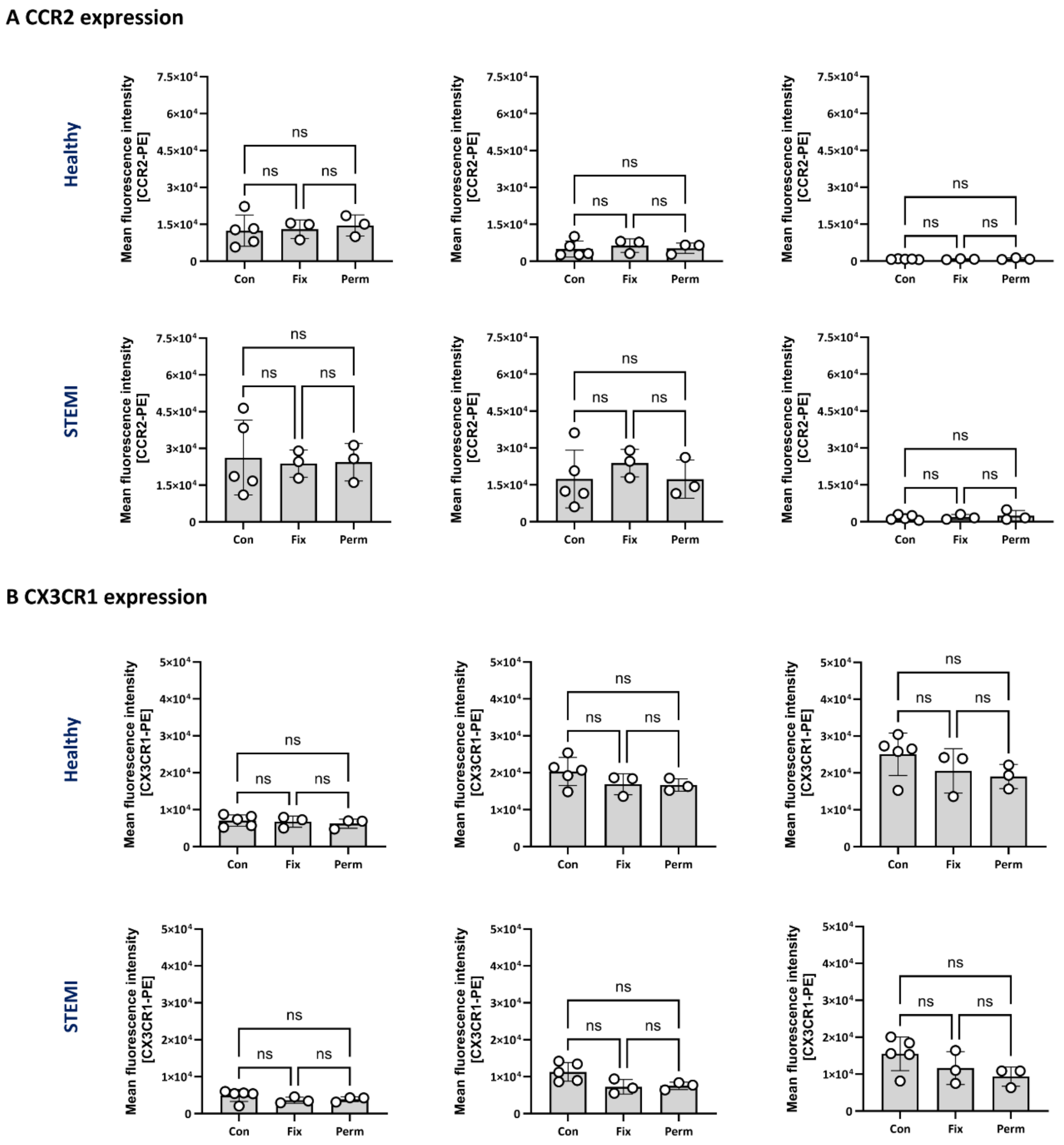

Figure S8: Expression of CCR2 and CX3CR1 in fixed and permeabilized monocytes: Cells derived from blood samples of healthy volunteers or patients on day 1 after ST-elevation myocardial infarction (STEMI) were stained with antibodies against CD11b, HLA-DR, CD14, CD16 (Strategy C) and CCR2 or CX3CR1. Subsequently, cells were left untreated (Con), fixed (Fix) or fixed and permeabilized (Perm) (see section 2.3.2 for details). Finally, cells were analyzed by flow cytometry and the expression of CCR2 **(A)** and CX3CR1 **(B)** (mean fluorescence intensity) was determined in monocyte subsets. Data show the mean fluorescence intensity ± SD of *n*= 3-5 individual samples. Gauss distribution was analyzed by Shapiro-Wilk test and then either a nonparametric Kruskal-Wallis test followed by post-hoc Dunn´s test or a one way ANOVA with a Turkey post-hoc analysis was performed.

Supplemental material

Supplementary Table S1: Information on the study groups

Experiment	Groups	N-number	Age (years)	Male/female
Fig. 1	Healthy Volunteers	11	22-31	3/8
Fig. 2	Healthy Volunteers	15	22-33	3/12
	Coronary heart disease (CHD)	10	52-86	5/5
	ST-elevation myocardial infarction (STEMI)	12	40-78	11/1
Fig. 3	Healthy Volunteers	5	22-27	1/4
	ST-elevation myocardial infarction (STEMI)	5	59-68	5/0
Fig. 4	Healthy Volunteers	9-11	22-27	2/9
Fig. 5 A	Healthy Volunteers	10	22-45	5/5
	ST-elevation myocardial infarction (STEMI)	10	41-80	8/2
Fig. 5 B	Healthy Volunteers	12	22-33	2/10
	Coronary heart disease (CHD)	10	52-86	5/5
	ST-elevation myocardial infarction (STEMI)	9	40-78	8/1
Fig. 6	Healthy Volunteers	5-6	22-33	2/4

Supplementary Table S2: Lists of antibodies

Strategy A				
**Specificity**	**Fluorochrome**	**Clone**	**Manufacturer**	**Dilution**
**CD11b**	APC	ICRF44	BioLegend GmbH (Fell, Germany)	1:200
**CD14**	PE-Cyanine7	63D3	BioLegend GmbH (Fell, Germany)	1:200
**CD16**	APC-Cyanine7	3G8	BioLegend GmbH (Fell, Germany)	1:200

Strategy B				
**Specificity**	**Fluorochrome**	**Clone**	**Manufacturer**	**Dilution**
**CD11b**	APC	ICRF44	BioLegend GmbH (Fell, Germany)	1:200
**CD14**	PE-Cyanine7	63D3	BioLegend GmbH (Fell, Germany)	1:200
**CD16**	APC-Cyanine7	3G8	BioLegend GmbH (Fell, Germany)	1:200
**CD3**	FITC	HIT3a	BioLegend GmbH (Fell, Germany)	1:200
**CD209**	FITC	9E9A8	BioLegend GmbH (Fell, Germany)	1:200
**CD19**	FITC	HIB19	BioLegend GmbH (Fell, Germany)	1:200

Strategy C				
**Specificity**	**Fluorochrome**	**Clone**	**Manufacturer**	**Dilution**
**CD11b**	APC	ICRF44	BioLegend GmbH (Fell, Germany)	1:200
**CD14**	PE-Cyanine7	63D3	BioLegend GmbH (Fell, Germany)	1:200
**CD16**	APC-Cyanine7	3G8	BioLegend GmbH (Fell, Germany)	1:200
**HLA-DR**	PerCP-Cyanine5.5	L243	BioLegend GmbH (Fell, Germany)	1:200

Intracellular staining (IC)				
**Specificity**	**Fluorochrome**	**Clone**	**Manufacturer**	**Dilution**
**CD11b**	APC	ICRF44	BioLegend GmbH (Fell, Germany)	1:200
**CD14**	PE-Cyanine7	63D3	BioLegend GmbH (Fell, Germany)	1:200
**CD16**	APC-Cyanine7	3G8	BioLegend GmbH (Fell, Germany)	1:200
**HLA-DR**	PerCP-Cyanine5.5	L243	BioLegend GmbH (Fell, Germany)	1:200
**CD74**	PE	LN2	BioLegend GmbH (Fell, Germany)	1:100
**HLA-DM**	PE	MaP.DM1	BioLegend GmbH (Fell, Germany)	1:100

CCR2, CX3CR1 and CD56 expression				
**Specificity**	**Fluorochrome**	**Clone**	**Manufacturer**	**Dilution**
**CD11b**	APC	ICRF44	BioLegend GmbH (Fell, Germany)	1:200
**CD14**	PE-Cyanine7	63D3	BioLegend GmbH (Fell, Germany)	1:200

**CD16**	APC-Cyanine7	3G8	BioLegend GmbH (Fell, Germany)	1:200
**HLA-DR**	PerCP-Cyanine5.5	L243	BioLegend GmbH (Fell, Germany)	1:200
**CCR2**	PE	K036C2	BioLegend GmbH (Fell, Germany)	1:200
**CX3CR1**	PE	K0124E1	BioLegend GmbH (Fell, Germany)	1:200
**CD56**	Brilliant Violet 421	5.1H11	BioLegend GmbH (Fell, Germany)	1:200

Supplementary Table S3: Abbreviations

2-NBDG	(2-(N-(7-Nitrobenz-2-Oxa-1,3-Diazol-4-yl)Amino)-2-Desoxyglucose)
**%**	Percent
**°C**	Degree celcius
**APC**	Allophycocyanin
**BSA**	Bovine serum albumin
**CCR**	C-C chemokine receptor
**CD**	Cluster of differentiation
**CHD**	Coronary heart disease
**CX3CR1**	CX3C motif chemokine receptor 1
**DAPI**	4′,6-diamidino-2-phenylindole
**EDTA**	Ethylenediaminetetraacetic acid
**e.g.**	Example given
**FACS**	Fluorescence activated cell sorting
**FBS**	Fetal bovine serum
**FITC**	Fluorescein isothiocyanate
**FSC**	Forward scatter
**g**	Gravity (9,81 m/s^2)
**h**	Hour
**HLA-DM**	Human leukocyte antigen DM
**HLA-DR**	Human leukocyte antigen DR
**IFN**	Interferon
**IgG**	Immunoglobulin G
**IL**	Interleukin
**Ly6c**	Lymphocyte antigen 6 complex
**LPS**	Lipopolysaccharide
**µg**	Microgram
**µl**	Microliter
**MFI**	Mean fluorescence intensity
**MHC**	Major histocompatibility complex
**ml**	Millilitre
**mM**	Millimolar
**NK-cells**	Natural killer cells
**nm**	Nanometre
**PBS**	Phosphate-buffered saline
**PCI**	Percutaneous coronary intervention
**PE**	Phycoerythrin
**PerCP**	Peridinin-chlorophyll-protein complex
**PFC**	Perfluorocarbon nanoemulsion
**RPMI**	Roswell Park Memorial Institute
**SSC**	Side scatter
**STEMI**	ST-elevation myocardial infarction
**TLR**	Toll-like receptors
**TNF**	Tumor necrosis factor
